# The design, implementation, and impact of an automated patient-reported outcome data collection and adverse event surveillance tool: a randomized trial

**DOI:** 10.1186/s12913-023-10231-1

**Published:** 2023-11-20

**Authors:** Megan S. Zhou, Tanya Jain, Nick Hardy, Alejandro Perez-Segura, Jasmine Hickman, Laurey Leopold, Kerry Qualliotine, Raagini S. Yedidi, Matthew Whetsell, Lauren Broffman

**Affiliations:** 1 Roman Health Ventures Inc, 116 W 23Rd St, New York, NY 10011 USA; 2 Two Sigma, New York, NY USA; 3 Garden City Hospital, Garden City, MI USA; 4 Big Whale Labs, New York, NY USA

**Keywords:** Patient-reported outcomes, Patient-reported experience measures, Healthcare quality improvement, Adverse events, Side effects, Telemedicine

## Abstract

**Background:**

Incorporating patient-reported outcome measures into routine clinical care can improve the patient experience, increase engagement, and establish a structured method for gathering adverse event (AE) data. Systematically collecting this information on a large scale can also inform new solutions for removing treatment barriers like medication nonadherence. This study evaluated whether implementing a patient-reported outcome data collection and adverse event surveillance tool would result in greater treatment continuation for patients receiving care on a telehealth platform.

**Methods:**

We used iterative plan-study-do-act cycles to evaluate how this data collection and surveillance tool—a short prompt for patients to provide information on treatment satisfaction and side effects—impacted treatment continuation, the outcome of interest. We tested two cycles in *n* = 2,000 patients receiving care for erectile dysfunction on a telehealth platform as a randomized controlled trial, and accounted for incidents where true randomization was not possible during implementation. The first cycle tested the tool alone, while the second cycle tested the tool in conjunction with a messaging template system that provided standardized side effect counseling.

**Results:**

Compared to patients in the control group, patients in the intervention group were more likely to refill their prescription over the duration of the study period (75% vs. 71%, Kaplan Meier log-rank test, *p* = 0.04). Receiving standardized counseling as part of the AE response system was positively associated with treatment continuation (*p* = 0.0005).

**Conclusions:**

Prompting patients to report side effects and outcomes outside of routine clinical visits has the potential to improve quality of care in virtual treatment.

**Trial registration:**

This trial has been retrospectively registered as a clinical trial (ClinicalTrials.gov Identifier: NCT05895539, registered June 8, 2023).

**Supplementary Information:**

The online version contains supplementary material available at 10.1186/s12913-023-10231-1.

## Background

In the past decade, there has been a significant shift towards patient-centered care, largely influenced by the provisions outlined in the Patient Protection and Affordable Care Act. This shift has expanded the use of patient-reported outcomes (PROs) beyond their traditional role in clinical research and integrated them into routine clinical care. PROs consist of information gathered directly from patients about their care experiences and the outcomes of their treatment [1]. Engaging patients by incorporating patient-reported outcome measures (PROMs) into routine clinical care has been shown to enhance the patient experience by improving communication between patients and providers, symptom management, patient satisfaction, and overall quality of life [2, 3].

One novel use for PROs is the assessment of adverse events [4]. Research suggests that systematically collecting data on side-effect related PROs can lead to improvement in clinician and patient communication [5]. However, the “collection of large-scale patient-reported adverse event (AE) data poses challenges for data capture, storage, security and integration into patient care pathways” [6]. Direct-to-consumer (DTC) platforms designed for large-scale operation and the systematic collection of structured data are well-equipped to address these challenges. They have the potential to offer innovative solutions to persistent obstacles in successful treatment, such as medication non-adherence. Non-adherence is a multifactorial problem; however, research shows that medication side effects are a driver [7], even though most side effects can be anticipated and discussed with the patient. Thus, DTC patients might benefit from the development of a centralized, automated process to encourage patients to report these experiences: a standardized process that allows providers to respond appropriately, and a systematic analysis to evaluate the impact of these interventions on quality of care.

As part of an internal quality improvement initiative, an interdisciplinary team at a DTC telehealth platform designed a PROM instrument intended to improve treatment continuation for patients. In response to concern that patients were discontinuing treatment due to manageable side effects experienced early in the course of their treatment, the care delivery quality and safety team identified the need to reach out to patients prior to their one-year follow-up visit. We provide a comprehensive overview of the development and deployment of a system for collecting adverse event-patient reported outcomes (AE-PRO) data and its corresponding response mechanism. Additionally, we describe the execution of a randomized controlled trial designed to assess the system's impact.

## Methods

The aim of this study was to implement a large-scale AE-PRO data collection and response system and evaluate how it affected treatment continuation and quality of care delivered on a DTC platform. This system's development was initiated in response to patient chart audits conducted by the care quality and safety team. These audits uncovered that certain patients were experiencing manageable side effects early in their treatment, leading them to discontinue it prematurely. Many of the remaining patients adjusted medication at the one-year follow-up visit included in usual care. Together, the quality and safety team and a technical infrastructure project team identified the need to proactively reach out to patients about side effect experiences earlier in the treatment course.

We used iterative Plan-Do-Study-Act (PDSA) cycles, a common quality improvement (QI) framework. The first PDSA cycle involved four steps: 1) the design of the data collection tool; 2) the pilot deployment of the tool to a randomly selected group of new patients; 3) the systematic assessment of whether the collection of AE-related PROs within the first few weeks of treatment would lead to better treatment outcomes; and, 4) the rollout of the tool platform-wide. The second cycle involved implementing a new intervention to assist providers in managing the increase in side effects reporting after the rollout of the form. This intervention entailed developing suggested messaging for providers to counsel patients around the most common mild (or combination of mild) side effects. Integrity of the data was maintained and checked by data analysts and members of the quality and safety team throughout the process of both cycles. Generalizability to healthcare settings external to the DTC platform was not evaluated. Age and geographic region but not sex were reported as descriptive demographic characteristics because the nature of the study population – those receiving care for erectile dysfunction – meant that the population consisted exclusively of male patients. States were categorized into geographic regions consistent with US Census Bureau designations, which can be seen in the Additional file 1: Appendix.

### Setting

This study took place on a DTC telehealth platform. Though the platform provides care for a variety of conditions to patients living in the US, we piloted our tool in those receiving care for erectile dysfunction. The procedure for accessing and receiving standard care through the platform involved the following steps:

1. The patient completed a dynamic online intake form that collected demographic information, health history, and other information relevant for diagnosis and to evaluate treatment appropriateness.
2. A provider then reviewed patient intake forms and, in some cases, completed synchronous telehealth visits with potentially eligible patients to determine and prescribe, if any, the appropriate course of treatment. Patients prescribed treatment also received a treatment plan that included information about side effects.
3. After their initial prescription and prior to one-year follow-up, the platform provided patients with access to an online messaging platform where they could communicate with providers at any time.

### Intervention design

#### Cycle 1

A crossfunctional team of technical leads and quality and safety staff collaborated to design a simple data collection tool that would be sent to new patients 14 days after initiating treatment. The intervention, referred to as an Rx Check-In (RxCI), was a short questionnaire that collected information from patients on treatment satisfaction and side effects. The RxCI additionally gauged patient interest in adjusting their medication to alleviate side effects and/or achieve greater medication efficacy. Information from patients requiring follow-up was automatically sent to providers so they could offer the necessary counseling and/or medication adjustment required. Those who did not require follow-up were also given the option to directly message their provider with any additional information via a chat interface. The purpose of the intervention was multifaceted: first, it was designed to facilitate better communication between patients and providers in the early stages of treatment. The intention was that better communication would lead to appropriate counseling and/or medication adjustment before patients abandoned treatment due to side effects or concerns around efficacy. A secondary purpose of the intervention was to facilitate systematic, structured data collection on rates of medication side effects that could be analyzed at the population level. A visual depiction of the full questionnaire is included in the Additional file 1: Appendix.

After the data collection tool was created, we decided to pilot the implementation of the tool with patients receiving treatment for erectile dysfunction (ED), a common male sexual dysfunction estimated to have affected more than 30 million American men [8]. There were several reasons why ED patients were the chosen population for piloting the data collection tool. American Urological Association (AUA) guidelines for the treatment of erectile dysfunction strongly recommend (evidence level grade B) that for men who are prescribed phosphodiesterase-5 (PDE-5) inhibitors, the dose should be titrated, defined as ongoing dose adjustment to provide optimal efficacy [9] with minimum adverse effects as mild side effects are common. Further evidence shows that switching from one type of PDE-5 inhibitor to another can help improve issues with efficacy and side effects [10]. Because medication adjustment is a process, patients need ongoing communication with providers after receiving their initial prescription. Without this, the combination of lack of requisite additional engagement and possible delay in achieving desired outcomes might contribute to low rates of medication adherence [11].

#### Cycle 2

To support providers in managing the increased volume of side effect reporting resulting from the cycle 1 intervention, the Quality and Safety team developed standardized messaging language for providers to use as a template in counseling patients around the most common mild (or combination of mild) side effects. These templates were intended to give providers a foundation from which to promptly and thoroughly respond to patient concerns. Reports of mild side effects were categorized by type and then aggregated across the pilot sample. The scripts including the messaging template tool were then developed based on the most frequently reported, or frequently reported combination, of side effects experienced by ED patients.

Providers were able to review responses to individual PROs collected via the data collection tool, and then quickly add templates into the chat application using simple keyboard shortcuts (e.g. “/headache”) that they were able to edit as needed. These templaces included more detailed, timely counseling for patients compared to the general information on side effects included in the treatment plan given to them at the onset of treatment as part of usual care. The team also developed a process to ensure prompt responses to increased reports of serious side effects. When a patient indicated a serious side effect on the form, a message was automatically delivered to an on-call nurse that was monitoring any communication. The nurse would then ensure appropriate patient follow-up.

In determining how to categorize the severity of side effects, mild side effects were defined as unwanted reactions to a drug that were not likely to result in death, permanent disability, or hospitalization, while serious side effects were defined as uncommon, unexpected, and/or severe reactions to a drug that might result in death, permanent disability, or hospitalization.

### Implementation process & timelines

#### Cycle 1

**Fig. 1:**
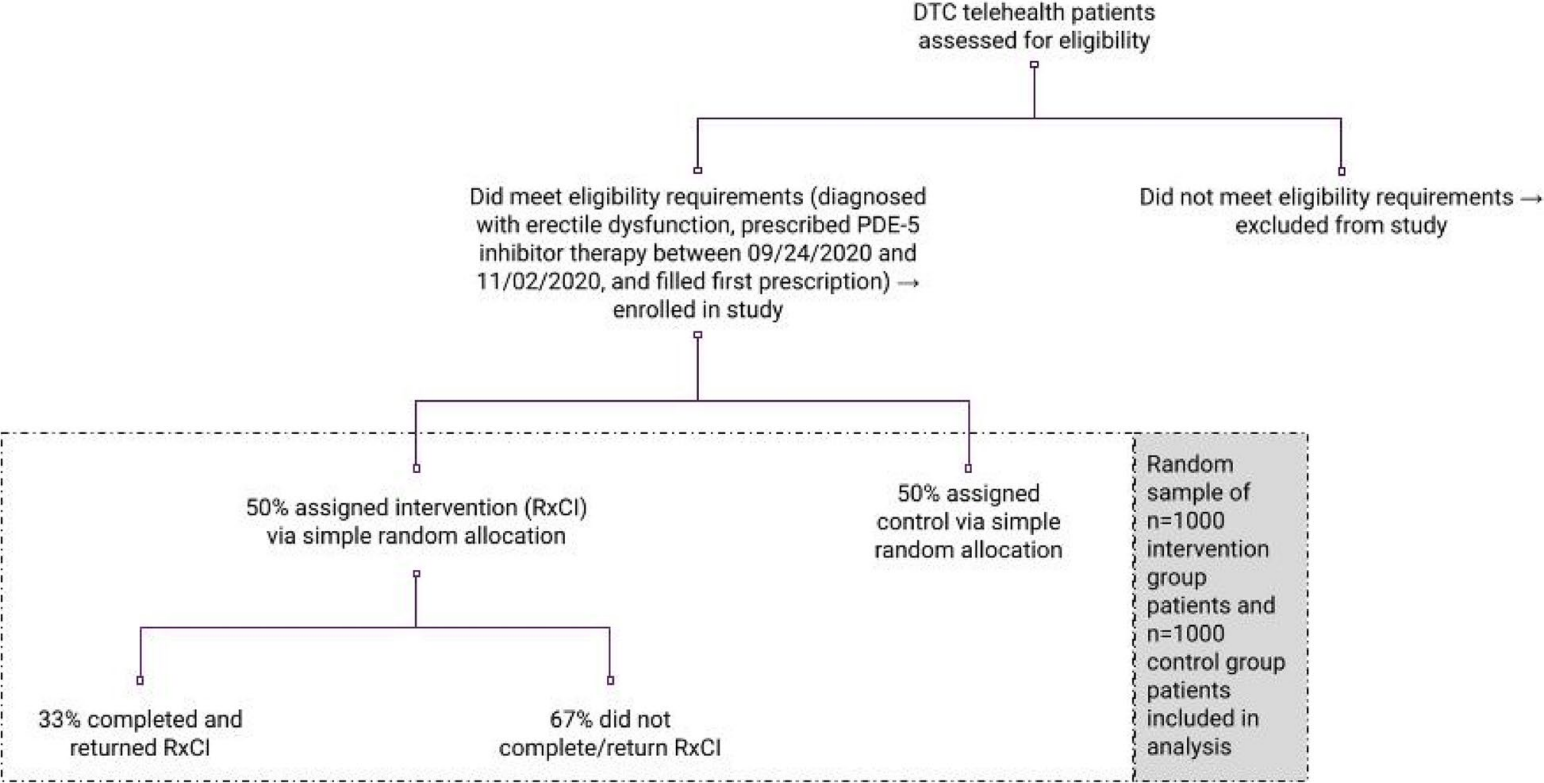
Patient flow

To investigate the effects of the data collection tool, we used an experimental design involving randomization. Patients were randomly assigned to either a control group that received standard care or an intervention group. The intervention group, alongside standard care, received the data collection tool two weeks after their initial prescription, using a parallel design with a 1:1 allocation (refer to Fig. 1). Participants were included in the trial if they were diagnosed with erectile dysfunction and prescribed PDE-5 inhibitor therapy between 09/24/2020 and 11/02/2020. The intervention was delivered randomly to 50% of these patients two weeks after their initial prescription, during the period of 10/08/2020—11/16/2020. The simple random allocation sequence was generated and implemented using computer software. Participants were automatically enrolled and assigned their intervention groups following randomization. A random sample of 1,000 patients in the intervention group and 1,000 patients in the control group was included in this analysis. Though no expected incidence information or literature on clinically meaningful differences existed for this specific type of intervention and context, the sample size was selected to ensure a robust but not overpowered analysis. Blinding participants was not possible due to the nature of the intervention.

#### Cycle 2

The team developed the counseling message templates by manually auditing patient charts, characterizing the most common mild side effects, and carefully writing the templates for accuracy and comprehensiveness of information using patient-centric language (e.g. ensuring readability, incorporating a conversational tone). These templates took several months to develop and were evaluated in a new cohort, in summer 2021.

### Evaluation methods

#### Cycle 1

Two outcomes were assessed in evaluating the data collection tool: 1) a pre-specified outcome of treatment continuation, defined as the placement and receipt of a prescription refill, and 2) a post-hot outcome of medication adjustment, defined as a change in medication type, quantity, and/or dosage at any point during their treatment plan. Treatment continuation was assessed within a specific time frame depending on the quantity of medication in their initial prescription. Patients were able to select either a monthly or quarterly (3-month) supply of medication. For monthly patients, treatment continuation was assessed within a time frame of 91 days. For quarterly patients, treatment continuation was assessed within a time frame of 123 days. These time frames were chosen in order to reflect individual and situational variation that may impact how often patients take their ED medication. All data was extracted from the telehealth platform’s electronic health record (EHR) database, where information collected from patients’ intake forms, telehealth visits, and other care-related interactions is stored.

Kaplan–Meier curves were used to examine unadjusted time-to-event treatment continuation. Due to the nature of the treatment continuation measure (placement and receipt of a prescription refill order), an “alive” event was used in place of the traditional death event. Patients were considered censored if there was no prescription refill within the specified timeframe. Log rank tests were used to determine statistical significance. Measuring the impact of the RxCI on treatment continuation served a dual purpose; it provided a secondary measure of patient uptake and tolerance of an additional point of contact. We used a chi square test of association to assess our post-hoc outcome of whether the proportion of patients who adjusted their treatment differed between groups. Lastly, we conducted an ancillary analysis using multivariable logistic regression to examine the effects of randomization into the intervention group. In this model, we employed medication adjustment as a control variable, along with age, to predict treatment continuation. An additional multivariable model including an intervention group-by-medication adjustment interaction term was also examined. To gauge the effectiveness of the intervention's implementation, we assessed the response rate to the questionnaire.

#### Cycle 2

Starting in June of 2021, the message template feature was made available to half of platform providers, selected at random. To assess the effectiveness of message templates, we examined the rates of treatment continuation among the 754 patients who reported a side effect and received counseling during that June. We compared the continuation rates between patients whose interactions with providers involved the use of message templates (*n* = 396) and those whose providers did not use them (*n* = 358). However, the rigorous methods as used in cycle 1 were undermined by contamination of the randomized rollout. In the care model, nurses “float” as needed, and sometimes work with doctors outside of their team. This meant that some nurses had access to the message templates when responding to individual side effect PROs for patients whose primary provider was supposed to be in the control group. Because of this, only chi squared tests of association were used to compare the treatment continuation of patients who received templated messages that continued treatment vs. those that did not.

All analyses for both cycles were conducted in R version 4.1.

## Results

In accordance with the patient population under study – those receiving care for erectile dysfunction – all patients were male. Age and geographic region were similarly distributed across control and intervention groups. Patients in the sample were, on average, middle-aged. The youngest patient was 18 years old, while the oldest was 87. Patients across both control and intervention groups were more likely to be between 30–59, with fewer patients in the younger and older age ranges. Most patients resided in the Southern region of the United States, followed by the West, Midwest, and Northeast (Table 1).

### Cycle 1

**Fig. 2:**
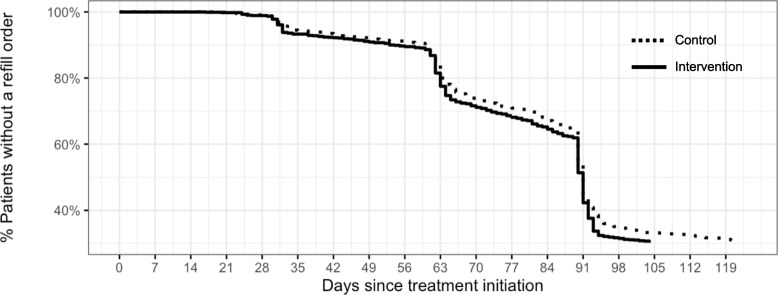
Unadjusted Kaplan–Meier survival curve for control and intervention patients

Approximately 33% of patients who received the RxCI responded and completed it. In the survival analysis estimating likelihood of prescription refill (Fig. 2), the Kaplan–Meier curves differed between the control and intervention groups; based on the log rank test, this difference was statistically significant (*p* = 0.04). By the end of the study period, 4% more of the intervention group than the control group had refilled their prescription; 75.2% (100 – 24.8%) of the intervention group refilled, while 71.1% (100 – 28.9%) of the control group refilled. Tables with the full Kaplan–Meier estimates for intervention and control groups are included in the Additional file 1: Appendix. A slightly higher proportion of the intervention group (5.4%) had their medication adjusted, compared to the control group (3.3%); results of the chi square test of association showed this difference was significant ($\chi^{2}$= 4.291, *p* = 0.038).

**Table 1:** Demographic characteristics (*n* = 2000)^a^

	Control (*n* = 1000)	Intervention (*n* = 1000)
Mean age, years (SD)	46 (13)	46 (14)
Age range, years	18—82	18—87
Age categories, % (n)		
18—29 years	12.5 (125)	13.1 (131)
30—44 years	34.5 (345)	34.0 (340)
45—59 years	36.4 (364)	34.5 (345)
60 + years	16.6 (166)	18.4 (184)
Geographic region, % (n)		
Northeast	15.5 (155)	18.8 (188)
Midwest	22.3 (223)	22.8 (228)
South	37.3 (373)	35.2 (352)
West	24.8 (248)	23.1 (231)
Missing^b^	(1)	(1)

^a^Sex is not reported, as only male patients can be prescribed ED medication through the telehealth platform. Regions are from US Census Bureau

^b^Those with missing data were not included in percentage calculations

After controlling for age and medication adjustment in a multivariable-adjusted logistic model, those who received the intervention (the RxCI questionnaire) had 1.21 times the odds (95% CI: 1.03, 1.44) of prescription refill as those in the control group (Table 2). Adjusting medication was associated with 3.18 times the odds (95% CI: 1.84, 5.99) of prescription refill, compared to those who did not adjust medication. There were no significant interactions between intervention group and medication adjustment (interaction OR 0.38, 95% CI: 0.08, 1.37). Age was not significantly associated with odds of prescription refill in any of the logistic regression analyses.

**Table 2:** Multivariable-adjusted effect of intervention on treatment continuation (*n* = 2000)

	Without interaction term		With interaction term	
Variable	OR	95% CI	OR	95% CI
Intervention group	1.21	(1.03, 1.44)	1.24	(1.04, 1.46)
Age	1.01	(1.00, 1.02)	1.01	(1.00, 1.02)
Medication adjustment	3.18	(1.84, 5.99)	6.12	(2.17, 26.58)
Intervention group^*^medication adjustment interaction	–	–	0.38	(0.08, 1.37)

*interaction term

Because of intervention group outcome superiority compared to control, the RxCI was delivered to all ED patients starting 11/17/2020, effectively ending the experimental period. No important harms or unintended effects were observed in either the intervention or control group. The tool was implemented in patient care for all other conditions, including mental health, weight management, herpes, and prescription dermatological treatments.

### Cycle 2

Out of the 754 patients who reported experiencing side effects in cycle 2, 96.7% of the 396 patients who received responses from their providers containing the messaging templates continued their treatment. Among the 358 patients whose responses did not include messaging templates, 90.2% continued their treatment. A chi squared test of association found the 6.5 percentage point difference was statistically significant ($\chi^{2}$= 12.23. *p* = 0.0005). No important harms or unintended effects were observed in either patients who received the templates or those who did not.

## Discussion

We found that our AE-PRO data collection and response system significantly increased treatment continuation over the duration of the study period. Though we were unable to assess the impacts of side effect counseling templates through a controlled experiment, the significant, positive correlation between template use and treatment continuation suggests potential for improving patient care, and warrants further research.

In cycle 1, the RxCI intervention led to an ultimate difference in treatment continuation of approximately four percentage points, compared to control (75% intervention group vs. 71% control group). This difference occurred regardless of whether patients in the intervention group fully engaged with the questionnaire (i.e. completed and returned it). It's possible that some patients in the intervention group who didn't return their questionnaires reached out to their providers directly. If this was the case, it implies that completing the questionnaire itself might not have been as critical as nudging patients to engage with their care. Given the technical infrastructure of the EHR data, we were unable to formally test this hypothesis but it remains an area for future research.

We additionally found that the effect of the RxCI intervention and of medication adjustment each independently affected the likelihood that patients refilled their next prescription. The lack of a statistically significant intervention-by-medication-adjustment interaction term implied that administering the intervention did not appear to strengthen the association between medication adjustment and prescription refill. However, the positive association between medication adjustment and prescription refill (regardless of intervention assignment), combined with the higher likelihood of prescription refill in intervention group patients, together suggest that medication adjustment played a role in treatment continuation, and that the RxCI provided a helpful channel through which patients could request such adjustment. While there are no existing studies evaluating the impact of PROMs on treatment continuation for ED patients, this study’s results corroborate previous findings that integrating PRO tools into routine care can help healthcare organizations assess adverse events [4] and improve patient outcomes in clinical settings [12], facilitating improved patient experience [2, 3]. We also found that providing standardized, comprehensive messaging templates for providers to counsel patients on mild side effects had a significant, positive association with treatment continuation in cycle 2. Treatment continuation was almost seven percentage points higher in patients who received messages that contained the counseling templates, compared to those who did not. Though we were not able to reproduce the same rigor of experimental testing for these templates, these results highlight the potential for standardized messaging and side effect mitigation to improve treatment outcomes.

The study’s primary limitations are 1) possible noise in the EHR data resulting from unlikely but plausible scenarios that would lead to measurement error, such as a prescription refill being the result of correcting an initial cancellation and not a true continuation of treatment, 2) the potential for nonresponse bias in the returning of the questionnaire, and 3) possible lack of generalizability beyond the specific telehealth platform and population in which the experiment was conducted. Limitations notwithstanding, we believe our findings provide a useful and illustrative starting point for other organizations seeking to implement similar AE-PRO collection and response systems. Though the study population consisted of patients receiving PDE-5 inhibitors, our findings may also apply more widely to medications that also require adjustment to achieve optimal efficacy and tolerability and thus greater medication adherence, but more research is needed to determine whether this is the case. Measuring treatment continuation in ED is challenging. Unlike life-sustaining medications for chronic conditions, most PDE-5 inhibitors are taken as needed, and prior research indicates that patient behavior as it relates to treatment continuation is subject to a number of factors, such as medication cost and the influence of sexual partners [11, 13]. However, by randomizing patients into intervention and control groups, these factors should “wash out,” allowing the attribution of any differences in groups to be the intervention. Study strengths also include its large sample size (2,000 patients) in initial testing, and its automated implementation, which reduces noise associated with inconsistent rollout that is more likely to occur in manual processes. Our findings show how digital health might have the ability to stimulate active patient engagement, leading to enhanced telecare quality. For digital healthcare companies with a national presence, establishing systems for collecting and responding to AE-PROs could yield substantial and diverse datasets regarding adverse events that could significantly contribute to the advancement of medical knowledge.

For monitoring and maintaining any increase in rates of treatment continuation and side effect reporting, consistent audits will be conducted, and necessary process improvements will be implemented. Our findings are a promising start, but iterative processes that test variations of both intervention protocols and the intervention itself might result in a more dramatic effect; for example, increasing the number of prompts or testing across different delivery vehicles (e.g. via text instead of email). It is presumed that treatment continuation leads to optimal patient outcomes, but future research can include clinical and quality of life outcome measures to test this hypothesis. Nonetheless, interventions that center patient-reported outcomes and experiences have the potential to improve the quality of care for patients receiving virtual treatment for erectile dysfunction and possibly other conditions in which side effects play a role in treatment continuation.

## Conclusions

Our findings indicate that prompting patients to report outcomes outside of routine clinical visits has the potential to improve quality of care for patients receiving virtual treatment. Interventions that leverage virtual platform capabilities to automatically collect patient-reported outcomes and provide comprehensive, standardized clinical counseling may further improve the quality of care for conditions in which side effects play a role in treatment continuation.

### Supplementary Information

**Additional file 1: Appendix.**

## Data Availability

The datasets generated and/or analyzed during the current study are not publicly available due to sensitivity of patient/human subjects data but may be available from the authors on reasonable request.

## Abbreviations

AE: Adverse event
ED: Erectile dysfunction
EHR: Electronic health record
OR: Odds ratio
PDE-5: Phosphodiesterase-5
PRO: Patient-reported outcome
PROM: Patient reported outcome measure
RxCI: Rx Check-In
