# Supplementary material for: The design, implementation, and impact of an automated patient-reported outcome data collection and adverse event surveillance tool: a randomized trial
Source: BMC Health Serv Res. 2023 Nov 20;23:1277. doi: 10.1186/s12913-023-10231-1 (PMC10658802; doi:10.1186/s12913-023-10231-1)
Supplement: Supplementary file 1 — Additional file 1: Appendix. [file 12913_2023_10231_MOESM1_ESM.docx]

**APPENDIX**

**Questionnaire**


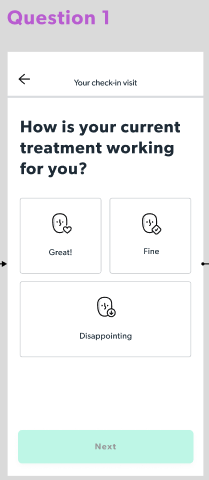


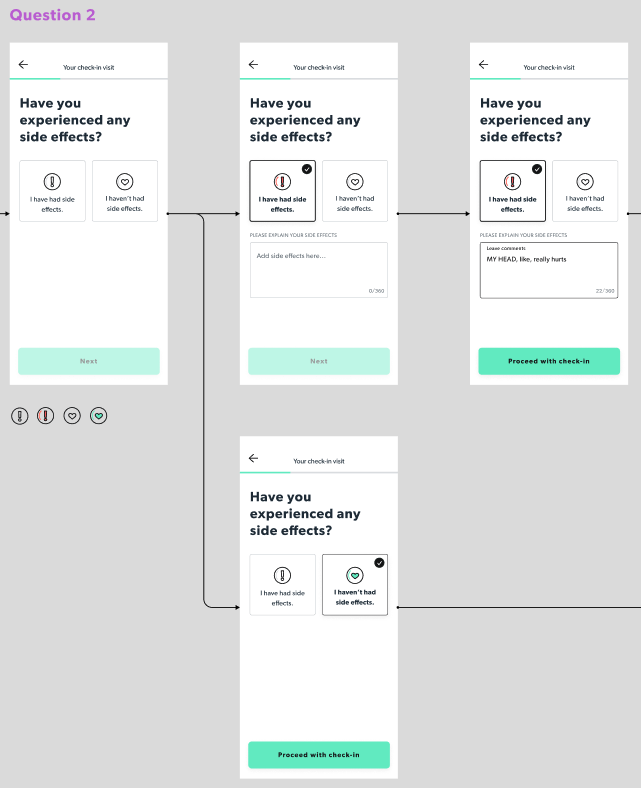


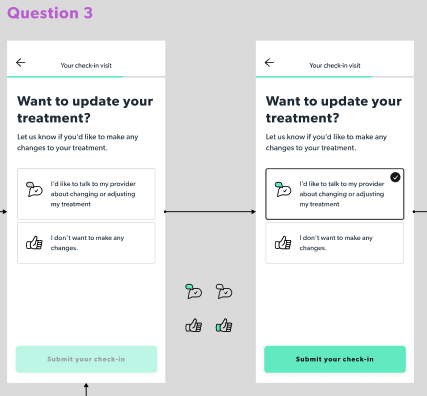


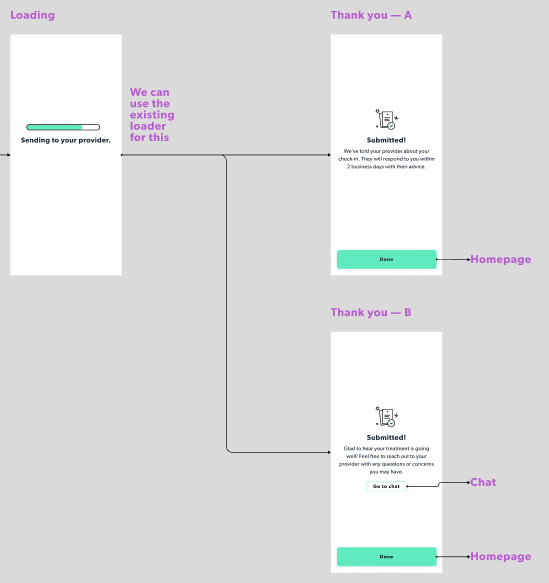


**Kaplan-Meier Estimates**

Table S1a. Kaplan-Meier estimates, control group

| Days since treatment initiation | Number at risk | Number of alive events | Proportion at risk | Standard error | Lower 95% CI | Upper 95% CI |
| --- | --- | --- | --- | --- | --- | --- |
| 19 | 1000 | 1 | 0.999 | 0.000999 | 0.997 | 1 |
| 22 | 999 | 2 | 0.997 | 0.001729 | 0.994 | 1 |
| 23 | 997 | 2 | 0.995 | 0.00223 | 0.991 | 0.999 |
| 25 | 995 | 2 | 0.993 | 0.002636 | 0.988 | 0.998 |
| 26 | 993 | 1 | 0.992 | 0.002817 | 0.986 | 0.998 |
| 27 | 992 | 1 | 0.991 | 0.002986 | 0.985 | 0.997 |
| 29 | 991 | 3 | 0.988 | 0.003443 | 0.981 | 0.995 |
| 30 | 988 | 4 | 0.984 | 0.003968 | 0.976 | 0.992 |
| 31 | 984 | 15 | 0.969 | 0.005481 | 0.958 | 0.98 |
| 32 | 969 | 12 | 0.957 | 0.006415 | 0.945 | 0.97 |
| 33 | 957 | 6 | 0.951 | 0.006826 | 0.938 | 0.964 |
| 34 | 951 | 6 | 0.945 | 0.007209 | 0.931 | 0.959 |
| 35 | 945 | 1 | 0.944 | 0.007271 | 0.93 | 0.958 |
| 36 | 944 | 1 | 0.943 | 0.007332 | 0.929 | 0.957 |
| 37 | 943 | 2 | 0.941 | 0.007451 | 0.927 | 0.956 |
| 38 | 941 | 1 | 0.94 | 0.00751 | 0.925 | 0.955 |
| 39 | 940 | 1 | 0.939 | 0.007568 | 0.924 | 0.954 |
| 40 | 939 | 2 | 0.937 | 0.007683 | 0.922 | 0.952 |
| 41 | 937 | 2 | 0.935 | 0.007796 | 0.92 | 0.95 |
| 42 | 935 | 3 | 0.932 | 0.007961 | 0.917 | 0.948 |
| 43 | 932 | 4 | 0.928 | 0.008174 | 0.912 | 0.944 |
| 44 | 928 | 2 | 0.926 | 0.008278 | 0.91 | 0.942 |
| 45 | 926 | 1 | 0.925 | 0.008329 | 0.909 | 0.941 |
| 47 | 925 | 1 | 0.924 | 0.00838 | 0.908 | 0.941 |
| 48 | 924 | 2 | 0.922 | 0.00848 | 0.906 | 0.939 |
| 49 | 922 | 2 | 0.92 | 0.008579 | 0.903 | 0.937 |
| 50 | 920 | 2 | 0.918 | 0.008676 | 0.901 | 0.935 |
| 51 | 918 | 2 | 0.916 | 0.008772 | 0.899 | 0.933 |
| 52 | 916 | 2 | 0.914 | 0.008866 | 0.897 | 0.932 |
| 53 | 914 | 2 | 0.912 | 0.008959 | 0.895 | 0.93 |
| 56 | 912 | 3 | 0.909 | 0.009095 | 0.891 | 0.927 |
| 57 | 909 | 1 | 0.908 | 0.00914 | 0.89 | 0.926 |
| 58 | 908 | 1 | 0.907 | 0.009184 | 0.889 | 0.925 |
| 59 | 907 | 2 | 0.905 | 0.009272 | 0.887 | 0.923 |
| 60 | 905 | 8 | 0.897 | 0.009612 | 0.878 | 0.916 |
| 61 | 897 | 18 | 0.879 | 0.010313 | 0.859 | 0.899 |
| 62 | 879 | 40 | 0.839 | 0.011622 | 0.817 | 0.862 |
| 63 | 839 | 39 | 0.8 | 0.012649 | 0.776 | 0.825 |
| 64 | 800 | 19 | 0.781 | 0.013078 | 0.756 | 0.807 |
| 65 | 781 | 15 | 0.766 | 0.013388 | 0.74 | 0.793 |
| 66 | 766 | 9 | 0.757 | 0.013563 | 0.731 | 0.784 |
| 67 | 757 | 5 | 0.752 | 0.013656 | 0.726 | 0.779 |
| 68 | 752 | 5 | 0.747 | 0.013747 | 0.721 | 0.774 |
| 69 | 747 | 8 | 0.739 | 0.013888 | 0.712 | 0.767 |
| 70 | 739 | 4 | 0.735 | 0.013956 | 0.708 | 0.763 |
| 71 | 735 | 4 | 0.731 | 0.014023 | 0.704 | 0.759 |
| 72 | 731 | 5 | 0.726 | 0.014104 | 0.699 | 0.754 |
| 73 | 726 | 2 | 0.724 | 0.014136 | 0.697 | 0.752 |
| 74 | 724 | 4 | 0.72 | 0.014199 | 0.693 | 0.748 |
| 75 | 720 | 6 | 0.714 | 0.01429 | 0.687 | 0.743 |
| 76 | 714 | 1 | 0.713 | 0.014305 | 0.686 | 0.742 |
| 77 | 713 | 4 | 0.709 | 0.014364 | 0.681 | 0.738 |
| 78 | 709 | 4 | 0.705 | 0.014421 | 0.677 | 0.734 |
| 80 | 705 | 7 | 0.698 | 0.014519 | 0.67 | 0.727 |
| 81 | 698 | 1 | 0.697 | 0.014532 | 0.669 | 0.726 |
| 82 | 697 | 10 | 0.687 | 0.014664 | 0.659 | 0.716 |
| 83 | 687 | 6 | 0.681 | 0.014739 | 0.653 | 0.711 |
| 84 | 681 | 10 | 0.671 | 0.014858 | 0.643 | 0.701 |
| 85 | 671 | 3 | 0.668 | 0.014892 | 0.639 | 0.698 |
| 86 | 668 | 9 | 0.659 | 0.014991 | 0.63 | 0.689 |
| 87 | 659 | 8 | 0.651 | 0.015073 | 0.622 | 0.681 |
| 88 | 651 | 2 | 0.649 | 0.015093 | 0.62 | 0.679 |
| 89 | 649 | 4 | 0.645 | 0.015132 | 0.616 | 0.675 |
| 90 | 645 | 96 | 0.549 | 0.015735 | 0.519 | 0.581 |
| 91 | 549 | 102 | 0.447 | 0.015722 | 0.417 | 0.479 |
| 92 | 447 | 38 | 0.409 | 0.015547 | 0.38 | 0.441 |
| 93 | 409 | 24 | 0.385 | 0.015387 | 0.356 | 0.416 |
| 94 | 385 | 17 | 0.368 | 0.01525 | 0.339 | 0.399 |
| 95 | 368 | 8 | 0.36 | 0.015179 | 0.331 | 0.391 |
| 96 | 360 | 4 | 0.356 | 0.015141 | 0.328 | 0.387 |
| 97 | 356 | 5 | 0.351 | 0.015093 | 0.323 | 0.382 |
| 98 | 351 | 3 | 0.348 | 0.015063 | 0.32 | 0.379 |
| 99 | 348 | 2 | 0.346 | 0.015043 | 0.318 | 0.377 |
| 100 | 346 | 3 | 0.343 | 0.015012 | 0.315 | 0.374 |
| 101 | 343 | 3 | 0.34 | 0.01498 | 0.312 | 0.371 |
| 102 | 340 | 5 | 0.335 | 0.014926 | 0.307 | 0.366 |
| 103 | 335 | 2 | 0.333 | 0.014903 | 0.305 | 0.364 |
| 105 | 333 | 1 | 0.332 | 0.014892 | 0.304 | 0.363 |
| 106 | 332 | 1 | 0.331 | 0.014881 | 0.303 | 0.361 |
| 107 | 331 | 2 | 0.329 | 0.014858 | 0.301 | 0.359 |
| 109 | 329 | 1 | 0.328 | 0.014846 | 0.3 | 0.358 |
| 110 | 328 | 1 | 0.327 | 0.014835 | 0.299 | 0.357 |
| 112 | 327 | 3 | 0.324 | 0.014799 | 0.296 | 0.354 |
| 113 | 324 | 2 | 0.322 | 0.014776 | 0.294 | 0.352 |
| 114 | 322 | 4 | 0.318 | 0.014727 | 0.29 | 0.348 |
| 115 | 318 | 1 | 0.317 | 0.014714 | 0.289 | 0.347 |
| 117 | 317 | 1 | 0.316 | 0.014702 | 0.288 | 0.346 |
| 118 | 316 | 2 | 0.314 | 0.014677 | 0.287 | 0.344 |
| 119 | 314 | 2 | 0.312 | 0.014651 | 0.285 | 0.342 |
| 120 | 312 | 2 | 0.31 | 0.014625 | 0.283 | 0.34 |
| 121 | 310 | 6 | 0.304 | 0.014546 | 0.277 | 0.334 |
| 122 | 304 | 8 | 0.296 | 0.014436 | 0.269 | 0.326 |
| 123 | 296 | 7 | 0.289 | 0.014335 | 0.262 | 0.319 |

Table S1b. Kaplan-Meier estimates, treatment group

| Days since treatment initiation | Number at risk | Number of alive events | Proportion at risk | Standard error | Lower 95% CI | Upper 95% CI |
| --- | --- | --- | --- | --- | --- | --- |
| 17 | 1000 | 1 | 0.999 | 0.000999 | 0.997 | 1 |
| 20 | 999 | 1 | 0.998 | 0.001413 | 0.995 | 1 |
| 24 | 998 | 5 | 0.993 | 0.002636 | 0.988 | 0.998 |
| 25 | 993 | 3 | 0.99 | 0.003146 | 0.984 | 0.996 |
| 26 | 990 | 1 | 0.989 | 0.003298 | 0.983 | 0.995 |
| 29 | 989 | 2 | 0.987 | 0.003582 | 0.98 | 0.994 |
| 30 | 987 | 9 | 0.978 | 0.004639 | 0.969 | 0.987 |
| 31 | 978 | 17 | 0.961 | 0.006122 | 0.949 | 0.973 |
| 32 | 961 | 23 | 0.938 | 0.007626 | 0.923 | 0.953 |
| 33 | 938 | 2 | 0.936 | 0.00774 | 0.921 | 0.951 |
| 34 | 936 | 3 | 0.933 | 0.007906 | 0.918 | 0.949 |
| 37 | 933 | 4 | 0.929 | 0.008122 | 0.913 | 0.945 |
| 38 | 929 | 1 | 0.928 | 0.008174 | 0.912 | 0.944 |
| 39 | 928 | 2 | 0.926 | 0.008278 | 0.91 | 0.942 |
| 40 | 926 | 2 | 0.924 | 0.00838 | 0.908 | 0.941 |
| 41 | 924 | 1 | 0.923 | 0.00843 | 0.907 | 0.94 |
| 42 | 923 | 1 | 0.922 | 0.00848 | 0.906 | 0.939 |
| 43 | 922 | 1 | 0.921 | 0.00853 | 0.904 | 0.938 |
| 44 | 921 | 2 | 0.919 | 0.008628 | 0.902 | 0.936 |
| 45 | 919 | 1 | 0.918 | 0.008676 | 0.901 | 0.935 |
| 46 | 918 | 3 | 0.915 | 0.008819 | 0.898 | 0.932 |
| 47 | 915 | 1 | 0.914 | 0.008866 | 0.897 | 0.932 |
| 48 | 914 | 3 | 0.911 | 0.009004 | 0.894 | 0.929 |
| 49 | 911 | 2 | 0.909 | 0.009095 | 0.891 | 0.927 |
| 50 | 909 | 2 | 0.907 | 0.009184 | 0.889 | 0.925 |
| 52 | 907 | 2 | 0.905 | 0.009272 | 0.887 | 0.923 |
| 53 | 905 | 5 | 0.9 | 0.009487 | 0.882 | 0.919 |
| 54 | 900 | 1 | 0.899 | 0.009529 | 0.881 | 0.918 |
| 55 | 899 | 2 | 0.897 | 0.009612 | 0.878 | 0.916 |
| 56 | 897 | 2 | 0.895 | 0.009694 | 0.876 | 0.914 |
| 58 | 895 | 3 | 0.892 | 0.009815 | 0.873 | 0.911 |
| 59 | 892 | 1 | 0.891 | 0.009855 | 0.872 | 0.911 |
| 60 | 891 | 5 | 0.886 | 0.01005 | 0.867 | 0.906 |
| 61 | 886 | 18 | 0.868 | 0.010704 | 0.847 | 0.889 |
| 62 | 868 | 53 | 0.815 | 0.012279 | 0.791 | 0.839 |
| 63 | 815 | 40 | 0.775 | 0.013205 | 0.75 | 0.801 |
| 64 | 775 | 28 | 0.747 | 0.013747 | 0.721 | 0.774 |
| 65 | 747 | 13 | 0.734 | 0.013973 | 0.707 | 0.762 |
| 66 | 734 | 6 | 0.728 | 0.014072 | 0.701 | 0.756 |
| 67 | 728 | 4 | 0.724 | 0.014136 | 0.697 | 0.752 |
| 68 | 724 | 2 | 0.722 | 0.014167 | 0.695 | 0.75 |
| 69 | 722 | 6 | 0.716 | 0.01426 | 0.689 | 0.745 |
| 70 | 716 | 5 | 0.711 | 0.014335 | 0.683 | 0.74 |
| 71 | 711 | 3 | 0.708 | 0.014378 | 0.68 | 0.737 |
| 72 | 708 | 6 | 0.702 | 0.014464 | 0.674 | 0.731 |
| 73 | 702 | 5 | 0.697 | 0.014532 | 0.669 | 0.726 |
| 74 | 697 | 4 | 0.693 | 0.014586 | 0.665 | 0.722 |
| 75 | 693 | 2 | 0.691 | 0.014612 | 0.663 | 0.72 |
| 76 | 691 | 5 | 0.686 | 0.014677 | 0.658 | 0.715 |
| 77 | 686 | 5 | 0.681 | 0.014739 | 0.653 | 0.711 |
| 78 | 681 | 3 | 0.678 | 0.014776 | 0.65 | 0.708 |
| 79 | 678 | 5 | 0.673 | 0.014835 | 0.645 | 0.703 |
| 80 | 673 | 2 | 0.671 | 0.014858 | 0.643 | 0.701 |
| 81 | 671 | 10 | 0.661 | 0.014969 | 0.632 | 0.691 |
| 82 | 661 | 6 | 0.655 | 0.015032 | 0.626 | 0.685 |
| 83 | 655 | 3 | 0.652 | 0.015063 | 0.623 | 0.682 |
| 84 | 652 | 7 | 0.645 | 0.015132 | 0.616 | 0.675 |
| 85 | 645 | 8 | 0.637 | 0.015206 | 0.608 | 0.668 |
| 86 | 637 | 5 | 0.632 | 0.01525 | 0.603 | 0.663 |
| 87 | 632 | 7 | 0.625 | 0.015309 | 0.596 | 0.656 |
| 88 | 625 | 2 | 0.623 | 0.015326 | 0.594 | 0.654 |
| 89 | 623 | 4 | 0.619 | 0.015357 | 0.59 | 0.65 |
| 90 | 619 | 105 | 0.514 | 0.015805 | 0.484 | 0.546 |
| 91 | 514 | 91 | 0.423 | 0.015623 | 0.393 | 0.455 |
| 92 | 423 | 47 | 0.376 | 0.015317 | 0.347 | 0.407 |
| 93 | 376 | 39 | 0.337 | 0.014948 | 0.309 | 0.368 |
| 94 | 337 | 13 | 0.324 | 0.014799 | 0.296 | 0.354 |
| 95 | 324 | 3 | 0.321 | 0.014763 | 0.293 | 0.351 |
| 96 | 321 | 2 | 0.319 | 0.014739 | 0.291 | 0.349 |
| 97 | 319 | 2 | 0.317 | 0.014714 | 0.289 | 0.347 |
| 98 | 317 | 2 | 0.315 | 0.014689 | 0.287 | 0.345 |
| 99 | 315 | 3 | 0.312 | 0.014651 | 0.285 | 0.342 |
| 100 | 312 | 1 | 0.311 | 0.014638 | 0.284 | 0.341 |
| 101 | 311 | 1 | 0.31 | 0.014625 | 0.283 | 0.34 |
| 102 | 310 | 2 | 0.308 | 0.014599 | 0.281 | 0.338 |
| 103 | 308 | 1 | 0.307 | 0.014586 | 0.28 | 0.337 |
| 104 | 307 | 5 | 0.302 | 0.014519 | 0.275 | 0.332 |
| 106 | 302 | 2 | 0.3 | 0.014491 | 0.273 | 0.33 |
| 108 | 300 | 2 | 0.298 | 0.014464 | 0.271 | 0.328 |
| 109 | 298 | 1 | 0.297 | 0.01445 | 0.27 | 0.327 |
| 110 | 297 | 3 | 0.294 | 0.014407 | 0.267 | 0.324 |
| 112 | 294 | 2 | 0.292 | 0.014378 | 0.265 | 0.322 |
| 113 | 292 | 1 | 0.291 | 0.014364 | 0.264 | 0.321 |
| 114 | 291 | 2 | 0.289 | 0.014335 | 0.262 | 0.319 |
| 115 | 289 | 4 | 0.285 | 0.014275 | 0.258 | 0.314 |
| 117 | 285 | 4 | 0.281 | 0.014214 | 0.254 | 0.31 |
| 118 | 281 | 1 | 0.28 | 0.014199 | 0.254 | 0.309 |
| 119 | 280 | 2 | 0.278 | 0.014167 | 0.252 | 0.307 |
| 120 | 278 | 3 | 0.275 | 0.01412 | 0.249 | 0.304 |
| 121 | 275 | 5 | 0.27 | 0.014039 | 0.244 | 0.299 |
| 122 | 270 | 10 | 0.26 | 0.013871 | 0.234 | 0.289 |
| 123 | 260 | 12 | 0.248 | 0.013656 | 0.223 | 0.276 |


Table S2. US Census Bureau Regions

| Region | States |
| --- | --- |
| Northeast | Connecticut, Maine, Massachusetts, New Hampshire, New Jersey, New York, Pennsylvania, Rhode Island, Vermont |
| Midwest | Kansas, Illinois, Indiana, Iowa, Michigan, Minnesota, Missouri, Nebraska, North Dakota, Ohio, South Dakota, Wisconsin |
| West | Alaska, Arizona, California, Colorado, Hawaii, Idaho, Montana, New Mexico, Nevada, Oregon, Utah, Washington, Wyoming |
| South | Alabama, Arkansas, Delaware, District of Columbia, Florida, Georgia, Kentucky, Louisiana, Maryland, Mississippi, North Carolina, Oklahoma, South Carolina, Tennessee, Texas, Virginia, West Virginia |
